# Supplementary material for: Whole blood transcriptional responses of very preterm infants during late-onset sepsis
Source: PLoS One. 2020 Jun 1;15(6):e0233841. doi: 10.1371/journal.pone.0233841 (PMC7263612; doi:10.1371/journal.pone.0233841)
Supplement: S4 Table — (DOCX) [file pone.0233841.s004.docx]

**Table S4. Differential cell count analysis between clinical groups.**

| **Cell Type** | **Confirmed LOS*** | **Possible LOS*** | **No LOS*** | **No LOS/Confirmed LOS**** | **No LOS/Possible LOS**** | **Confirmed LOS/Possible LOS**** |
| --- | --- | --- | --- | --- | --- | --- |
| White Cell Count  (x10^9^/L) | 20.20  (9.40 – 39.33) | 15.71  (3.36 – 20.59) | 18.10  (11.27 – 27.84) | 0.36 | 0.33 | 0.29 |
| Platelet Count  (x10^9^/L) | 118.00  (47.00 – 308.00) | 284.50  (123.00 – 475.00) | 372.00  (148.00 – 600.00) | 0.01 | 0.41 | 0.19 |
| Red Cell Count  (x10^12^/L) | 3.67  (2.65 – 4.15) | 3.15  (2.67 – 3.80) | 3.51  (2.70 – 4.59) | 0.90 | 0.33 | 0.41 |
| Neutrophil Count  (x10^9^/L) | 12.73  (3.20 – 29.10) | 8.94  (0.94 – 12.53) | 9.09  (4.06 – 17.26) | 0.44 | 0.50 | 0.19 |
| Lymphocyte Count  (x10^9^/L) | 5.45  (2.16 – 6.87) | 4.27  (1.92 – 6.59) | 5.79  (3.58 – 7.78) | 0.61 | 0.20 | 0.56 |
| Monocyte Count  (x10^9^/L) | 1.66  (0.28 – 6.47) | 1.17  (0.07 – 2.88) | 1.93  (0.79 – 4.18) | 0.90 | 0.33 | 0.56 |
| Eosinophil Count  (x10^9^/L) | 0.28  (0.00 – 1.08) | 0.34  (0.18 – 0.82) | 0.33  (0.18 – 0.38) | 0.97 | 0.64 | 0.96 |

*Median (Minimum – Maximum)

**The p-value was based on non-parametric Mann-Whitney test.
