# Supplementary material for: Whole blood transcriptional responses of very preterm infants during late-onset sepsis
Source: PLoS One. 2020 Jun 1;15(6):e0233841. doi: 10.1371/journal.pone.0233841 (PMC7263612; doi:10.1371/journal.pone.0233841)
Supplement: S5 Table — (DOCX) [file pone.0233841.s005.docx]

**Table S5. Genes associated with over-represented pathways identified using the Sigora gene-pair signature method.**

| **Pathway** | **Genes** |
| --- | --- |
| Activation of BAD and translocation to mitochondria | YWHAH;YWHAG;BID;YWHAZ;BMX;KIF14;TNFSF10;DTNBP1;NECAP1;SEC23A;CPD;SEC24A;STAM2;SORT1;SEC24D;AP3S1;COPB2;ACTR3;NCKAP1L;ABI1;ACTR2;ARPC5;VPS4B;CHMP2B;CYBB;NSL1;NUF2;MCM10;COPB1;GNS;CASC5;AP1G1;CLTC;LIMK2;CENPF;PAFAH1B1;MAPRE1;LMNB1;CTNNB1;CDC42;CCNA2;LY96 |
| Activation of BH3-only proteins | YWHAH;YWHAG;BID;YWHAZ;RAB10;STXBP3;RAB14;RHOQ;XIAP;CASP7;SNAP23;KIF3B;CEP162;EXOC5;EXOC6;RHOB;NBN;RHOA;PPP1R12A;RAB11A;ACTB |
| Activation of gene expression by SREBF (SREBP) | IDI1;SQLE;DHCR7;HMGCS1;MTF1;SREBF2;SMARCD3 |
| Alternative complement activation | CFD;C3;LAMP2;HGF;TF;PLEK;SRGN;CAP1;CD63;SERPING1;SERPINF2;CFL1;HSPA5;THBS1;IGF2 |
| Aquaporin-mediated transport | GNB1;GNG2;AQP9;VIPR1;PDE7B;RGS6;MLX;TMED5;RAB11FIP2;LAMP2;PLEK;SRGN;SOX4;CD63;DACT1;RAB11A;FGR;CCRL2;CAP1;SERPINF2;CX3CR1;PRKAR1A;PRKAR2A;GNG5;HGF;TF;STXBP3;CDC73;SERPING1;KREMEN1;CFD;ECT2;MCF2L;FGD4;LRP5;IGF2;DHRS9;XIAP;CFL1;PTPN1;THBS1;FCER1G;LDLR;HSPA5;MYC;LYN;ACTB;GSK3B;LAMTOR3;KBTBD7;KRAS;HIST1H2AC;HIST1H2BK;SPRED2 |
| Cell surface interactions at the vascular wall | CD58;MERTK;CD177;CEACAM1;SIRPA;SLC16A3;ITGAM;FCER1G;ITGB2;LYN;KRAS;CD44;PIK3CB |
| Cholesterol biosynthesis | DHCR24;LBR;IDI1;SQLE;DHCR7;HMGCS1 |
| Clathrin derived vesicle budding | GNS;DTNBP1;CPD;SCARF1;MARCO;APOL1;CD163;HP;PGD;HSP90B1;IDH1;PFKFB2;SLC25A12;PGM2;PGAM1;TPMT;HK3;MMADHC;UGP2;TCN2;CYP1B1;TBXAS1;SLC2A3 |
| Constitutive Signalling signalling by NOTCH1 HD Domain Mutants | ADAM17;FAS;FUZ;TULP3;CEACAM1;EPS15;ULK3;TMED2;MFNG;IQCE;MFAP1;FBLN2;PAG1;FKBP1A;ITGA7;ICAM1;TGFBR1;STAM2;CDC73;RALB;ITGAM;KREMEN1;PTCH1;IFT140;NEDD4;ITGB2;TNFSF10;CASK;PTPN1;STAT1;THBS1;SOCS3;TCF7L2;E2F3;SOCS1;CDC42;DUSP3;LYN;MAPK14 |
| Dectin-2 family | CLEC4A;CLEC4E;CLEC4D;FCER1G |
| Diseases of immune system | NFKBIA;TLR2;MYD88;TLR5;IRAK4;CHUK;IRAK3;SOCS1;TBK1;TLR8;IRF7;MAP3K8;S100A12;MAP2K6;UBE2D1;RIPK2;DUSP3;MAPK14;IRAK2 |
| Hedgehog "on" state | PTCH1;ULK3;FUZ;TULP3;IFT140 |
| Interferon alpha/beta signalling | IFI35;IFIT3;IFIT2;XAF1;RNASEL;IFITM3;IFITM1;MX1;OAS1;OAS3;GBP2;ISG15;SOCS1;IRF7 |
| Interferon gamma signalling | GBP3;GBP1;MT2A;TRIM5;TRIM22;GBP5;GBP4;IFNGR1;ICAM1;OAS1;OAS3;GBP2;SP100;CD44;IRF7;SOCS1 |
| Interferon Signalling | EIF4G3;EIF4E3;IFI35;IFIT3;IFIT2;XAF1;IFITM3;NEDD4;RNASEL;GBP3;GBP1;MT2A;TRIM5;TRIM22;GBP5;GBP4;STAT1;IFITM1;UBE2E1;IFNGR1;MX1;SP100;ICAM1;CD44;JAK2;OAS1;OAS3;GBP2;IRF7;SOCS3;SOCS1 |
| Interleukin-1 signalling | IL1R2;IL1R1;IL1RN;IL1B;IRAK3 |
| Interleukin-6 signalling | SOCS3;UBA6;IFI35;UBE2A;UBE2W;WSB1;RNF19B;GBP3;GBP1;IFIT3;IFIT2;MT2A;TRIM5;TRIM22;XAF1;RNF138;RNASEL;RNF144B;IFITM3;GBP5;GBP4;DTX3L;TRIM69;IFITM1;UBE2J1;PJA2;ICAM1;NEDD4;FBXO6;MX1;SP100;UBA3;RCHY1;CD44;ISG15;UBE2D1;UBE2E1 |
| KSRP (KHSRP) binds and destabilizes mRNA | YWHAZ;KIF14;DTNBP1;NECAP1;SEC23A;BMX;CPD;SEC24A;STAM2;COPB1;SORT1;CDC73;SEC24D;AP3S1;KREMEN1;COPB2;ACTR3;NCKAP1L;ABI1;ACTR2;ARPC5;VPS4B;DCP2;LIMK2;CHMP2B;HCK;TNFSF10;CYBB;NSL1;NUF2;MCM10;GNS;CENPF;CASC5;AP1G1;LMNB1;CLTC;PAFAH1B1;MAPRE1;PIK3CB;GSK3B |
| Membrane Trafficking | COPB2;STAM2;SEC23A;SEC24A;SEC24D;RAB10;RAB14;VPS4B;DTNBP1;NECAP1;CPD;SORT1;AP3S1;CHMP2B;STXBP3;RHOQ;KIF3B;EXOC5;EXOC6;RAB11A;GNS;CLTC;AP1G1;SNAP23 |
| Metabolism of vitamins and cofactors | SLC2A3;MMADHC;PC;TCN2 |
| MyD88-independent TLR3/TLR4 cascade | UBE2D1;NFKBIA;RIPK2;MAP3K8;DUSP3;MAPK14;IRAK2;LY96;MYD88;UBA6;UBE2A;UBE2W;WSB1;RNF19B;RNF138;RNF144B;DTX3L;TRIM69;UBE2J1;PJA2;IL1B;ETS2;ITGB2;ITGAM;NEDD4;RCHY1;RNF2;FCER1G;CCNE2 |
| MyD88:Mal cascade initiated on plasma membrane | IRAK3;SOCS1;MAP3K8;S100A12;MAP2K6;RIPK2;DUSP3;LY96;MAPK14;IRAK2 |
| Negative regulators of RIG-I/MDA5 signalling | DDX58;UBE2D1;ISG15;TNFAIP3;TBK1;IFIH1;GBP3;GBP1;MT2A;TRIM5;TRIM22;GBP5;GBP4;FAS;SMAD1;IFNGR1;NLRC4;AIM2;IFI35;ICAM1;IFIT3;IFIT2;XAF1;IFITM3;FANCA;SP100;CLEC4A;SOD2;ATG3;PRDX3;CLEC4E;CLEC4D;OAS1;OAS3;MSH3;RNASEL;GBP2;IFITM1;CD44;TLR5;TLR8;RAB2A;NCAPH;RAB1A;HSP90B1;S100A12;MYD88;FKBP1A;NFKBIA;ERO1A;CNEP1R1;JAK2;TNFSF10;LIG4;BRCA2;TGFBR1;KIF23;PTPN1;HSBP1;XRCC4;SOCS3;IRF7;RFWD2;NBN;SOCS1;SEC23A;SEC24A;SEC24D;MCM10;CHMP2B;CYBB;CHUK;CTNNB1;RRAGD;RRAGC;YWHAH;CDC25A;RHOA;YWHAZ;YWHAG;PIK3CB;ACTB;CSF2RB;JAK3;KBTBD7;RAP1B;PTEN;SPRED2 |
| O-linked glycosylation of mucins | GALNT3;GALNT1;GALNT2;GALNT14;C1GALT1C1;B3GNT5;GCNT1;MUC1;B3GNT2;ST6GALNAC3;B4GALT5 |
| Oxygen-dependent proline hydroxylation of Hypoxia-inducible Factor Alpha | EGLN1;EPAS1;HIF1A;UBE2D1;SOD2;ATG3;PRDX3;ETS2;IGFBP7;CEBPB;HSBP1;RNF2;CHMP2B;HSPA5;CCNE2;RRAGD;NBN;RRAGC;LMNB1;MAP2K6;CCNA2;UBE2E1;MAPK14;HIST1H2AC;HIST1H2BK;LAMTOR3;E2F3;TMED2;ERO1A;SEL1L;CXCL8;GSK3B |
| Platelet degranulation | LAMP2;HGF;PLEK;SRGN;CAP1;CD63;SERPING1;CFL1;HSPA5;THBS1 |
| PPARA activates gene expression | HMGCS1;SLC27A1;PPARG;MED4;PLIN2;CPT2;NPAS2;MTF1;SREBF2;ALAS1;FADS1;ACSL1 |
| Regulation of cholesterol biosynthesis by SREBP (SREBF) | INSIG1;SEC23A;SEC24A;IDI1;SQLE;DHCR7;MTF1;HMGCS1;SREBF2;SMARCD3 |
| RHO GTPases activate WASPs and WAVEs | ACTR3;NCKAP1L;ABI1;ACTR2;ARPC5;CDC42 |
| Sema4D in semaphorin signalling | CDC42;LIMK2;RHPN1;KIF14;ARAP2;EPS15;RHOU;ARHGAP29;FAM13A;CDC42EP3;SRGAP3;ARHGAP11A;EFNB1;AR;RHOQ;NCKAP1L;ABI1;BAIAP2;YWHAH;CFL1;ARHGAP10;CCND3;STAM2;CYBB;NSL1;NUF2;CENPF;CASC5;ECT2;MCF2L;FGD4;PAFAH1B1;MAPRE1;CTNNB1;FCER1G;MYC;MAP3K8;YWHAZ |
| Signalling by Interleukins | IL1R2;IL1R1;IL1RN;HGF;HCK;SOCS3;IL1B;YWHAZ;KBTBD7;SPRED2;ACTB;LAMTOR3;PIK3CB;CSF2RB;JAK3;JAK2;IRAK3;KRAS;LYN;IRAK4;MAP3K8;IRAK2;MAP2K6;RIPK2;MYD88;CHUK |
| Signalling to RAS | ACTB;JAK2;KRAS;CSF2RB;LAMTOR3;KBTBD7;SPRED2;MKNK1;IFNGR1;RAB10;ACTR3;STXBP3;RAB14;RHOQ;NCKAP1L;ABI1;ACTR2;ARPC5;SOCS3;PTPN1;SNAP23;KIF3B;EXOC5;EXOC6;RAB11A;SOCS1;STAT1;NSL1;NUF2;RHOB;CENPF;CTNNB1;YWHAH;CASC5;YWHAZ;RHOA;PAFAH1B1;MAPRE1;CDK1 |
| Toll Like Receptor 3 (TLR3) cascade | TBK1;IRF7;UBE2D1;NFKBIA;RIPK2;DUSP3;MAP2K6;IRAK2;LY96;S100A12;MYD88;CHUK;ZBP1;IRAK3;SOCS1;TLR2;IRAK4 |
| Toll Like Receptor 9 (TLR9) cascade | NFKBIA;MAP3K8;DUSP3;MAP2K6;IRAK2;S100A12;MYD88;CHUK |
| Toll Like Receptor TLR1:TLR2 cascade | MAPK14;MAP2K6;CHUK;SOCS1;NFKBIA;RIPK2;MAP3K8;DUSP3;IRAK2;LY96;IRAK4;NEO1;BNIP2;BCL10;IFNGR1;GNA15;GNAQ;CTNNB1;CDC42;MKNK1;RHOA;GNB1;GNG5;GNG2 |
| Toll Like Receptor TLR6:TLR2 cascade | MAP2K6;RIPK2;MAPK14;CHUK;IRAK2;IRAK4;MAP3K8;NFKBIA;LY96;IL1R2;IL1R1;IL1RN;ITGB2;ITGAM;DUSP3;TLR2;IL1B;TNFAIP3;RTN4;ETS2;IGFBP7;CEBPB;ECT2;FGD4;RALB;FGR;ELMO2;PAG1;CXCL8;BMX;GNA13;LMNB1;BID;IFIH1;NCKAP1L;ABI1;UBA3;RHOA;NBN;CCNE2;BCL10;FCER1G;CASP7;ALAS1;PTEN;DCP2;XIAP;DDX58;APH1B;ADAM17;CYBB;YWHAZ;GSK3B;STAT1;PIK3CB;SMARCD3;PDE1B;UBE2E1;YWHAH;GNAI3 |
| Translocation of GLUT4 to the plasma membrane | RAB10;STXBP3;RAB14;RHOQ;SNAP23;KIF3B;EXOC5;EXOC6;RAB11A |
| TRIF-mediated TLR3/TLR4 signalling | TBK1;IRF7;UBE2D1;MAP3K8;S100A12;MAP2K6;RIPK2;DUSP3;MAPK14;IRAK2;LY96 |
| Vesicle-mediated transport | SCARF1;CD163;MARCO;APOL1;HP;HSP90B1;RAB10;RAB14;COPB1;COPB2;NECAP1;SNAP23;STAM2;STXBP3;SORT1;AP3S1;RHOQ;VPS4B;EXOC5;SEC23A;KIF3B;SEC24A;EXOC6;CHMP2B;RAB11A;AP1G1;YWHAH;CLTC;YWHAZ;YWHAG;ACTB |
