# Supplementary material for: Whole blood transcriptional responses of very preterm infants during late-onset sepsis
Source: PLoS One. 2020 Jun 1;15(6):e0233841. doi: 10.1371/journal.pone.0233841 (PMC7263612; doi:10.1371/journal.pone.0233841)
Supplement: Attachment — Submitted filename: PONE-D-19-34983 Response to Reviewers-AC_SN.docx [file pone.0233841.s006.docx]

**Whole blood transcriptional responses of very preterm infants during late-onset sepsis**

Sherrianne Ng^1^, Tobias Strunk^2^, Amy H. Lee^3,4^, Erin E. Gill^3,4^, Reza Falsafi^3,4^, Julie Hibbert^2^, Robert E. W. Hancock^3,4^ and Andrew Currie^1,2,*^

# Reviewer #1

In this manuscript, Dr. Ng and Colleagues used RNAseq to interrogate the whole blood response in preterm neonates with and without late-onset sepsis (LOS). The group has published several excellent studies on neonates, immunology, inflammation, and sepsis. There have been several studies of this type (whole blood RNA, neonate, sepsis) but to my knowledge this is the first to describe using RNAseq. They concluded host immune transcriptional responses that reflect unbalanced immunometabolic homeostasis.

As the authors are well aware, synthesis of neonatal sepsis studies is impaired by the lack of a consensus definition for sepsis in this population (PMID: 24751791).

There is a severity of illness score specific to preterm neonates with LOS that might be beneficial to include at the time of sepsis evaluation for the patients described herein (PMID: 31394566) so that the results can be interpreted in the context of future studies.

We acknowledge the lack of consensus definition for sepsis, which is a major limitation to current neonatal sepsis investigations. Accordingly, we have used a definition of confirmed sepsis that relies on both positive blood culture AND elevated CRP as marker of inflammation, in order to maximise the specificity of diagnosis for comparisons between sepsis and non-sepsis infants.

The development of the nSOFA score is an important advance in neonatal sepsis research, and one that we are now adopting for our current and planned sepsis studies, Unfortunately, we did not collect all of the required clinical data in the submitted study at the time of completion, so we are unable to calculate an nSOFA score for the infants included herein.

The median birth GA of the patients was very similar across groups but the birth weights were substantially different. Would the authors comment on this?

The difference observed in birth weights based on classification (i.e. Confirmed LOS, Possible LOS and No LOS) were mainly due to the limited number of samples within each group that resulted in skewed distributions and variability observed within each classification group. However, Although the median between groups appeared substantially different, there were no significant differences based on Mann-Whitney test (p>0.05), albeit with the limitations of sample size.

Would it be correct to assume no neonate died with the sepsis episode? Would it also be true that those without LOS received <=48 hrs abx? For infants that were in the possible LOS group - would it be true that they all received treatment for >5 days similar to those with a positive blood culture?

Although we do not have information with regards to time of death in relation to sampling time, infant 15106 died with the sepsis episode. Unfortunately, we do not have abx data available for all infants without LOS, however, for infants we have data for, some received <5 days of abx. The infants with possible LOS received varying durations of abx ranging from 3 – 8 days.

In the methods under RNAseq there is a statement that the samples were pooled. Later in the results there is a description of individual results. This is confusing and would benefit from clarification. If pooled, pooling may come with limitations that perhaps should be in the discussion (PMID: 26208977).

We have clarified the methods used for RNAseq in the methods section to state “To derive the transcriptome for each infant, samples were labelled with a unique barcode, multiplexed, and sequenced on a HiSeq 2500 sequencer (Illumina), using the High Output 100-bp single-end run, at the University of British Columbia Sequencing Centre. The deconvoluted sequences from each infant were analysed and the quality of FASTQ reads were assessed using FastQC(v0.0.15), and summarized using MultiQC(v0.8.dev0) [20, 21].” (line 109 – 114, page 6). Thus, while the samples were multiplexed for sequencing purposes, deconvoluted data was used to identify the transcriptomes of individual infants.

Some pathogens isolated from blood are unusual based on published large epidemiologic studies. Do the authors suspect that these unusual pathogens may have affected the applicability of the results?

All 5 pathogens identified in blood in our population are known to be associated with LOS (PMID: 25425653) and therefore, we believe our preliminary results will be informative and applicable to future studies investigating sepsis, particularly when examining pathogen responses at a class and genus level. However, we do acknowledge that larger studies are needed to examine species-specific differences in septic responses.

The types of cells in whole blood can modify the transcriptome. Were CBC assessments done for clinical reasons in proximity to the RNA assessments that might inform the analysis?

We thank the reviewer for this suggestion, and we have now extracted and analysed the CBC data for the infants in this study. The raw data for differential counts is provided with in Table S1 and descriptive statistics shown in Table S4. There was no significant differences in cell counts between the sepsis groups.

Consider including weeks of post-conceptual age alongside days on pg9 (line 158).

We have added the postmenstrual age into Table 1, page 8.

The data presented on pg 10 lines 163-168 is a bit confusing (confirmed v no sepsis = 1317 genes; confirmed v possible = 21 genes; possible v no sepsis = no differences). Would the authors clarify how the possible sepsis group can be so similar to both the confirmed bacteremic group and the no sepsis group?

We have addressed this in lines 441 - 459 on page 20 - 21.

IL-7 may indeed be beneficial to reduce adaptive impairment in sepsis. However a study of neonatal mice that lacked an adaptive immune system showed no difference in sepsis survival (PMID: 18591384) suggesting the role of adaptive immunity may be different in neonates compared to adults.

We do not believe that these findings detract from our observations and discussion regarding IL-7 and adaptive immunity in neonatal sepsis. The cited study relates to polymicrobial sepsis in a term neonate animal model which may be very different from the setting in human preterm infants in our study. We agree that the requirements for adaptive immunity may be different in neonates and adults, and add that differences may also exist with different neonatal groups. Clearly, further studies of adaptive immune responses (and Treg activity) are warranted in human neonatal sepsis.

The discussion is very thorough but perhaps a bit long.

We have taken this onboard and trimmed the discussion, where possible.

# Reviewer #2

The article by Ng et al. is interesting and important as it covers a much understudied area of developing next-generation biomarkers for sepsis in most at-risk neonates and deciphering potential causal molecular pathways using relatively unbiased approached.

However, the article has two major weaknesses that I think are absolutely essential to address in order to bring it to sufficient publication quality for PLoSOne:

1) Much of the discussion is based on differentially expressed genes, based on data that are not presented in the manuscript! Or at least it could possible that the list of differentially expressed genes (including estimates and adjusted significance measures) have been included with the submission but in any case I could not see these data. It is not sufficient to say that there were so many differentially expressed genes between groups, or that this or that gene was differentially expressed. The data has to be presented quantitatively. Please make sure it is included in the next submission.

We have included the data pertaining to differentially expressed genes identified between infants with confirmed LOS and no LOS (Table S2, line 167, page 9) and between infants with confirmed LOS and possible LOS (Table S3, line 169, page 9).

2) The manuscript goes on at length on negative findings, ignoring that this study is of extremely small sample size. Therefore, though I agree that the findings are original and worthy of publication the entire discussion has to be revised with considering this major limitation (I acknowledge that they list this as a limitation at the end of the discussion but they don’t sufficiently take this into consideration in their interpretation of data). Three examples:
a. Page 15, lines 291-293: the fact that infants with possible LOS had no differentially expressed genes compared to infants without LOS at the transcriptional-level may very well due to underpower and thus cannot be a major finding. To have this statement be a major finding would imply at least proper demonstration of power.

We have now addressed the low power due to the small sample size of our study and removed or toned down statements on negative findings in the discussion.

b. Same for the extended discussion around the lack of difference in IL-1b which may follow a trend but is clearly underpowered.

This has now been addressed - see page 16.

c. Page 20, lines 402: The possibility that many genes may not show up as differentially expressed needs to be considered here.

This has now been addressed - see page 20.

In addition, I have a number of other suggestions for improvement (ordered by appearance in text):

Introduction :

“Neonatal sepsis results in more than 450,000 infant deaths globally each year.” Since these are only gross estimates, based on extrapolated data, I think it should be reworded. Consider including a more recent reference than the Lawn J, such as this one latest one: KE Rudd, Lancet 2020 who report a potential for 801,615 neonatal deaths per year due to sepsis. Probably the “real” number is between half million to one million neonatal deaths due to sepsis each year.

Have included this recent reference and reworded the manuscript accordingly - see Page 3.

“In addition, preterm infants have also shown impairments in cellular metabolism that limit 40 innate immune responses during infection [11].” Please clarify. I think you mean that immune cells from preterm infants, not the preterm infants themselves…”.

We have clarified this in the manuscript - see page 3.

“However, no study to date has specifically investigated host transcriptional responses during LOS in very preterm infants, in whom the incidence of sepsis is highest.” “Our findings are the first to characterise the underlying functional biology of blood leukocyte transcriptional responses associated specifically with LOS immunopathology in very preterm infants. “This is not entirely true as the author point out the study by Cernada was conducted in VLBW infants. Please adjust these 2 statements and better emphasize how this study builds on Cernada’s findings in the introduction. This is crucial to increase the value of the authors’ work relatively to what has already been published.

We have changed our statement to clarify how our study adds to existing studies by being the first to use transcriptome sequencing (RNA-Seq) to investigate not only immune responses but immuno-metabolic responses of very preterm infants during LOS – see page 3. Where possible, we have also showed overlap in differentially expressed genes identified in our study with those in the study by Cernada (see pages 19 and 20).

Methods:

Please indicate the recommended blood culture volume and methods for infants in the study NICU and if available, how much blood was actually sent to the lab for infants in the study. Please also indicate what CRP assay was used in the study NICU at the time of the study period.

We have included the following statement into the methods: “Study patients born very preterm (n=20) were retrospectively classified, based on blood culture results using a recommended minimum volume of 0.5 mL for infants <28 weeks GA and plasma CRP measurements (Vitros, Ortho-Clinical Diagnostics)” – see page 4. We did not collect the blood volume sent to the lab for infants in this study, however, will endeavour to do so in future studies.

The rationale for the fold-change cut-off is unclear to me. Given the small dataset I would have only used an adjusted p value cut-off (the fold-change probably adds little and is somewhat arbitrary).

We included fold-change as a cut-off as this has been used in a previous transcriptional study of preterm and term infants with bacterial infections (PMID: 25120092) when determining differentially expressed genes to focus on.

Results:

Please provide the clinical data in non-aggregated form for all subjects in 3 group (not just septic babies) in a supplementary table, This will be crucial to allow future linkage by study ID to the transcriptome data submitted to GEO and help build on the author’s finding in future research.

We have included this data in Table S1, page 7.

The authors need to provide a list of differentially expressed genes with adjusted p values and effect size, between each group comparisons, in order to substantiate the core of their findings.

We have included data related to differentially expressed genes in Table S2 and Table S3 (page 9).

Can the author provide data on the blood count levels for the infants at the time of blood sampling for RNA?

We have included the raw data for differentially blood count levels in Table S1 and the descriptive statistics for comparisons between clinical groups in Table S4.

Discussion:

There are several comments made about the lack of secretion cytokines in these infants, although for cytokines like interferons (alpha or gamma) there is very little published report that these are sufficiently detectable levels in human blood and this may very well be due to the fact that these cytokines act locally in their micro-environment rather than systemically. I think the choice of measured cytokines need to be better justified and the comment on the lack of detection tone down, OR there needs to be strong literature support of the feasibility of detecting these cytokines, for example, in adults with sepsis (as a sort of “historical” positive control).

Where possible, we have toned down or removed findings for cytokines measured that lacked detection. The cytokines were chosen based on the gene-pair signaling pathways involved in cytokine signalling that were identified as significally over-represented (Table 2, pages 10 – 11) to determine whether changes at the transcriptional-level would be associated with downstream changes at the protein level.

Page 17, lines 338-339: In addition to my comment above, about the lack of detectable IL-1b and justification for measuring this cytokine (for interpretation of negative finding). I wonder if the authors have considered that the alternative explanation that the lack of IL-1b (assuming it is a detectable cytokine) could be related to a weak cytokine response predisposing preterm infants to sepsis rather than an active inhibition via IL1R2/IL1RN.

In lines 425 – 427 (page 20), we have highlighted weak IL-1b responses could also “be due to low expression of *NLRP3* in very preterm infants, where NLRP3 has a central role in regulating inflammasome activation and subsequent IL-1b secretion [37,97].”

I find the discussion around metabolism (particularly cholesterol but also basic energy metabolism) quite relevant and interesting. Therefore I suggest brining that up in the discussion and emphasizing this point. As the authors point out there is an exciting literature in adults with sepsis related to cholesterol metabolism for prognostication, the use of lipid proteins inducers and role in neutralization of LPS.

We have moved this to point 2 instead of point 3 of discussion.

SOCS1/SOCS3: without seeing the actual data (quantitative measures) it is very difficult to appreciate this section which seems a lot more speculative (in view also with the strong possibility that IFN may not be measurable meaningfully in blood).

We have provided the differentially expressed gene list in Table S2 (page 9). Briefly, between Confirmed LOS and No LOS infants, SOCS1 (3.38 fold-change, p=0.00058) and SOCS3 (2.47 fold-change, p= 0.019034604).

It would be informative to provide blood counts for these infants. In addition, the authors could estimate these counts using hematopoietic cell-specific markers. The possibility that differences in cell population is not sufficiently considered in the interpretation of the data. Estimating blood counts using in silico methods would strongly help such interpretation of the data where relevant.

We agree and we have provided the actual counts in Table S1 (page 9) as well as descriptive statistics between clinical groups in Table S4 (page 9) and agree that in silico counts will extend interpretation and we are not conducting a study to correlate. There is no validation of CIBERSORT with neonatal cells and separately pursuing as separate analysis. Too small a study to do it with.

Page 20, lines 402-417: There is a major obvious consideration that is missing here in this part of the discussion, which is the fact that among babies with possible sepsis there is likely a number that actually don’t have bacterial sepsis, but have non-bacterial sepsis or other inflammatory syndromes. Have the authors tried to apply their top differentially expressed genes from the confirmed sepsis group to try to separate this group further. This may be futile given the small sample size. At least discuss this possibility. At the moment, the paragraph is quite speculative in my view and the lack of power needs to be better considered. Were there any genes that were very close to the adjusted p value threshold? Impossible to say without seeing the data and an estimation of power needs to support the currently exposed speculations.

We are not able to determine as the adjusted p-value cut-off was 0.05 to identify differentially expressed genes. Future studies with a larger sample size could analyse the differentially expressed genes within the possible LOS group. We have however discussed the possibility that due to the small sample size of the possible LOS group, this could impact on the lack of differentially expressed genes identified (pages 20 – 21).
